# Factors Associated with Energy Efficiency of Focused Ultrasound Through the Skull: A Study of 3D-Printed Skull Phantoms and Its Comparison with Clinical Experiences

**DOI:** 10.3389/fbioe.2021.783048

**Published:** 2021-12-10

**Authors:** Chanho Kong, So Hee Park, Jaewoo Shin, Hee Gyu Baek, Juyoung Park, Young Cheol Na, Won Seok Chang, Jin Woo Chang

**Affiliations:** ^1^ Department of Neurosurgery, Brain Research Institute, Yonsei University College of Medicine, Seoul, South Korea; ^2^ Medical Device Development Center, Daegu-Gyeongbuk Medical Innovation Foundation, Daegu, South Korea; ^3^ Department of Neurosurgery, Catholic Kwandong University College of Medicine, International St Mary’s Hospital, Incheon, South Korea

**Keywords:** focused ultrasound, 3D print, skull factor, skull density ratio, blood-brain barrier, energy transmission

## Abstract

While focused ultrasound (FUS) is non-invasive, the ultrasound energy is attenuated by the skull which results in differences in energy efficiency among patients. In this study, we investigated the effect of skull variables on the energy efficiency of FUS. The thickness and density of the skull and proportion of the trabecular bone were selected as factors that could affect ultrasound energy transmittance. Sixteen 3D-printed skull models were designed and fabricated to reflect the three factors. The energy of each phantom was measured using an ultrasonic sound field energy measurement system. The thickness and proportion of trabecular bone affected the attenuation of transmitted energy. There was no difference in the density of the trabecular bone. In clinical data, the trabecular bone ratio showed a significantly greater correlation with dose/delivered energy than that of thickness and the skull density ratio. Currently, for clinical non-thermal FUS, the data are not sufficient, but we believe that the results of this study will be helpful in selecting patients and appropriate parameters for FUS treatment.

## Introduction

Since the introduction of ultrasound in the early 20th century, many neurosurgeons have tried to apply this noninvasive modality to treat central nervous system (CNS) diseases (Heimburger, 1958). However, the clinical applications of ultrasound for CNS diseases have been limited because the skull interferes with efficient energy transmission. In the 1990s, the phased array technique was developed, which allowed for the focusing of ultrasound through the skull and increased the possibility of applying ultrasound for CNS diseases (Hynynen et al., 1993). The first trial of focused ultrasound (FUS) for functional diseases was started in 2010, and accurate lesions were successfully made in the target area, demonstrating the potential of transcranial ultrasound application (McDannold et al., 2010).

Thermal FUS currently has clinical efficacy for the treatment of essential tremors (Chang et al., 2015; Elias et al., 2016; Weintraub and Elias, 2017), Parkinson’s disease (PD) (Na et al., 2015) (Bond et al., 2017) (Martinez-Fernandez et al., 2018) (Zaaroor et al., 2018), and obsessive-compulsive disorder (Jung et al., 2018; Jung et al., 2015; Kim et al., 2018) with a very low rate of permanent complications, and it is attracting attention as a noninvasive, alternative modality for CNS diseases. Meanwhile, research on non-thermal FUS for blood brain barrier (BBB) opening and neuromodulation has also been widely expanded (Hynynen et al., 2003; Treat et al., 2007; Vykhodtseva et al., 2008) (Tufail et al., 2010). BBB opening by FUS with microbubbles has already been verified through numerous preclinical studies, enabling the delivery of drugs that do not normally pass the BBB (Zhan, 2020). Recently, non-thermal FUS has been applied in clinical trials for brain tumors (Park et al., 2021), PD (Gasca-Salas et al., 2021), and Alzheimer’s disease (D’Haese et al., 2020; Lipsman et al., 2018), but it is still in the early stages of safety verification.

The skull is still the main obstacle to the delivery of uninterrupted ultrasonic energy to the brain’s target sites. While ultrasonic energy is transmitted through the skull, the energy is attenuated by being refracted and reflected by various skull variables (Chang et al., 2019; Chang et al., 2016; D’Souza et al., 2019). Based on failed attempts in which the temperature did not rise well for thermal ablation with FUS, the concept of the skull density ratio (SDR), which affects ultrasonic energy transmission, has emerged (Chang et al., 2015; Chang et al., 2016). By using this for patient selection, the efficiency of the procedure has been significantly improved.

It is thought that factors act differently for thermal and non-thermal FUS. To evaluate various skull-related variables affecting low-intensity ultrasonic energy transfer, skull phantoms of various conditions were made using a 3D printer. Furthermore, we reviewed the chart of patients who underwent BBB opening with magnetic resonance-guided FUS (MRgFUS) and analyzed the clinical data.

## Materials and Methods

### Experimental Study

#### 3D-Printed Model

**FIGURE 1:**
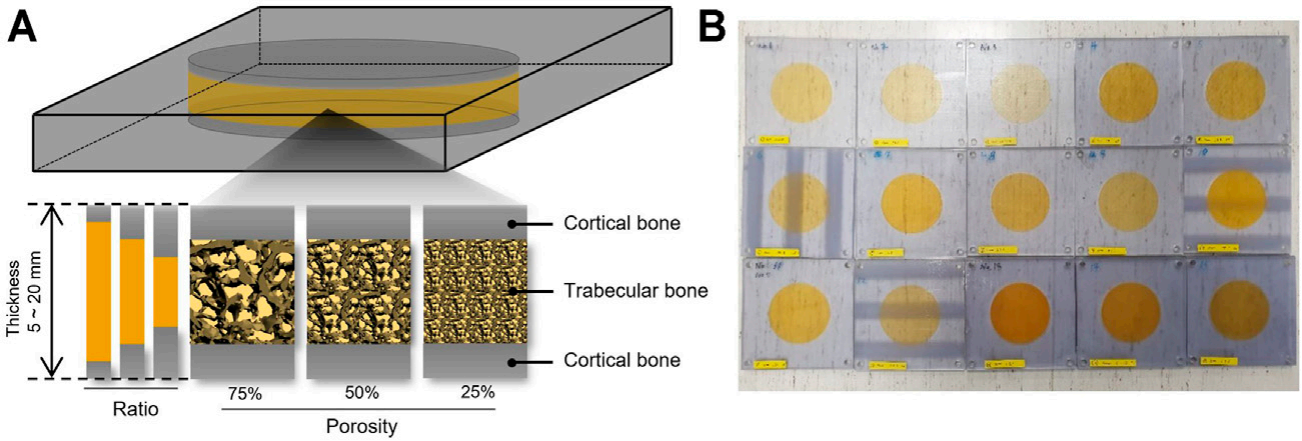
A schematic representation of skull 3D phantom. **(A)** Skull phantoms were designed and fabricated to reflect thickness, porosity, ratio of cortical bone and trabecular bone. **(B)** Fifteen skull phantoms were made with a 3D printer.

The 3D-printed skull phantoms were printed on a PolyJet-type 3D printer (Objet30 Prime, Stratasys, Edina, MN) at MEDYSSEY (Jecheon, Korea). A skull phantom was produced in the form of a dipole. The skull phantom (W180 and H180 mm) was made in the shape of a square, and the inner structure imitating the trabecular bone was designed as a circle with a diameter of 100 mm. The overall bone was simulated using MED 610, and the detailed structure of the trabecular bone was supported using SUP 706, a water-soluble support material. Biocompatible MED 610 is used in medical and dental applications where precise visualization and patient contact are required, and is a suitable material for simulating detailed structures with 3D printers. A study using a skull phantom made of MED 610 has been reported, and the acoustic properties of this material do not deviate significantly from the actual skull (Acquaticci et al., 2019) (Robertson et al., 2017). Phantoms were made by spraying liquid material and hardening and laminating through ultraviolet. A total of 16 skull phantoms were designed and fabricated by varying the thickness and density of the skull and the volume of the trabecular and cortical bones as factors (Table 1). A thickness of 10 mm was used as a reference, and phantoms of 5 and 20 mm thickness were made. The density of the trabecular bone was set at 25 and 75% based on a porosity of 50% (Alexander et al., 2019). The ratio of the trabecular bone to cortical bone was 1:8:1, 2:6:2, and 3:4:3 (Figure 1).

**TABLE 1:** Conditions of sixteen 3D printed skull models (C: cortical bone, T: trabecular bone).

Phantoms	Thickness (mm)	Ratio C/T/C (mm)	Porosity (%)
1	5	0.5/4/0.5	50
2	5	1/3/1	50
3	5	1.5/2/1.5	50
4	10	1/8/1	25
5	10	2/6/2	25
6	10	3/4/3	25
7	10	1/8/1	50
8	10	2/6/2	50
9	10	3/4/3	50
10	10	1/8/1	75
11	10	2/6/2	75
12	10	3/4/3	75
13	20	2/16/2	50
14	20	4/12/4	50
15	20	6/8/6	50

#### Ultrasound Transmit Measurement

**FIGURE 2:**
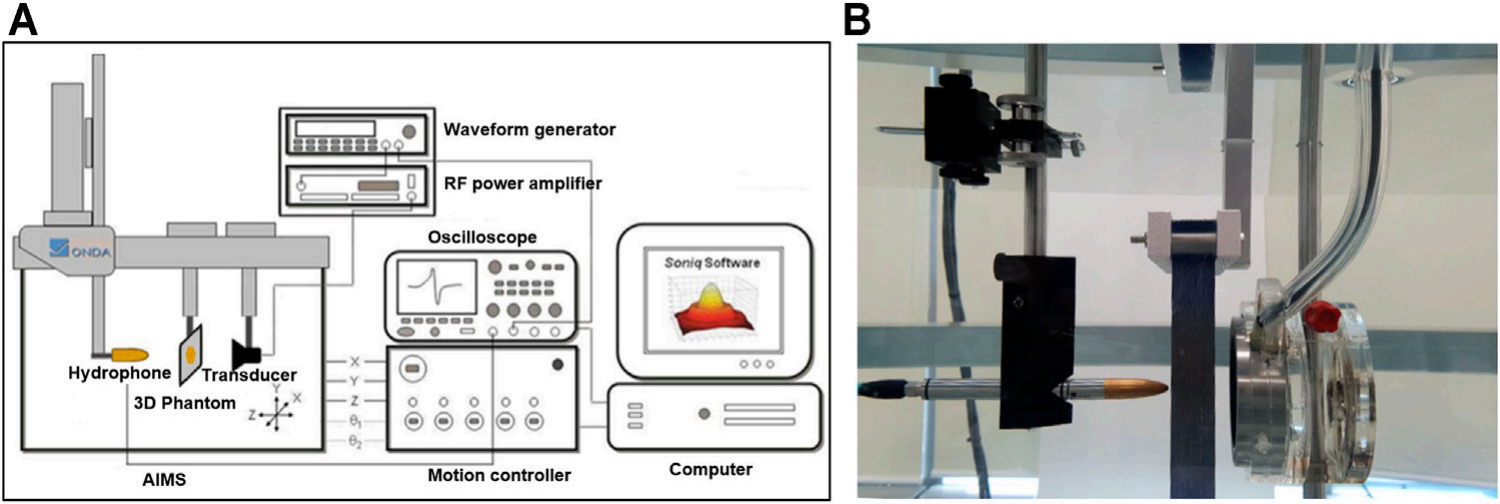
**(A)** The Acoustic Intensity Measurement System (AIMS) is a hydrophone scanning system that enhances acoustic measurement productivity to map acoustic fields in degassed water. **(B)** Measurement of acoustic pressure in the water tank of AIMS.

All measurements were performed in an acrylic tank filled with degassed water of the Acoustic Intensity Measurement System (AIMS III, ONDA, Sunnyvale, CA, United States). To measure the ultrasound pressure transmitted through the phantoms, a hydrophone (HGL-400, ONDA, Sunnyvale, CA, United States) was placed in the focal region of the transducer and the phantom was placed between the transducer and hydrophone (Figure 2). A pulsed ultrasound was generated using a single element-focused transducer (0.25 MHz, 64 mm diameter, and 63.2 mm radius curvature; H-115 model, SonicConcepts, Bothell, WA, United States). The excitation signal was initiated by the waveform generator (33220A, Agilent, Palo Alto, CA, United States) which is amplified through the Radio Frequency (RF) power amplifier (240L, E&I, Rochester, NY, United States) and then sent to the transducer. The acoustic fields were explored in two ways (1D and 2D scans), and the transmitted ultrasonic energy pressure was measured three times repeatedly. The peak pressure value was averaged, and the attenuation ratio was calculated using the peak pressure value without phantom.

### Clinical Data

#### Data Selection

We reviewed the data of 37 clinical study data in 8 glioblastoma patients who underwent BBB opening with MRgFUS using the ExAblate 4000 low-frequency device (230 kHz, Insightec, Ltd.) from August 2018 to March 2021 (IRB no. 1-2018-0040, clinical trial gov Identifier. NCT03712293). Sonication for BBB opening was performed with intravenous injection of microbubbles (Definity^®^; 4 μl/kg per single injection, maximum number of injections was 5). The optimal power of BBB opening is established as 50% of the power level at which bubble activity is high enough to generate inertial cavitation by ramp sonication. Once the power was established, a 90 s long sonication was performed, and the power was adjusted while monitoring the bubble activity during the sonication. The targets were selected around the tumor, and sonication of 1 cm^3^ was performed at the targets. BBB opening was confirmed in 92.0% of patients using postoperative T1-enhanced magnetic resonance imaging.

#### Skull-Related Variables

The skull-related variables were obtained using preoperative computed tomography (CT) images. SDR was calculated using the CT Density Analysis Tool (InSightec). Bone volumes were measured with the Aquarius iNtuition program (Version 4.4.11, TeraRecon, Foster City, CA, United States) using the Hounsfield unit (HU). By moving the HU window by 100 units and comparing this with the area of the bone above the anterior commissure (AC)-posterior commissure (PC) line in CT, the minimum and maximum values of the HU range of individual skull bones were set.

The criteria for dividing the cortical and trabecular bones were determined using HU. The volume of bone per 100 HU between the minimum and maximum HU was measured, and the different criteria were used to divide the cortical and trabecular bones according to the shape of the volume histogram. When looking at a volume histogram, there are three types of distribution: bimodal or left or right skewed. The dividing criteria applied to each type were as follows.

In the bimodal shape, when the two peak values are located on the left and right sides of the median of the peak values, respectively, the division criteria were defined as the HU value having the lowest volume between the two peak values. If not, the division criteria were set to the HU point with the largest slope from the peak value close to the median value of the minimum and maximum HU.

Even if the histogram has a bipeak shape, it was not considered a peak if the volume of the peak was less than 5% of the total volume, because it was judged to be less than the value evenly distributed. In the case of a skewed shape with one peak having a volume of 5% or more, the trabecular and cortical bones were divided at the point where the slope is the largest in the curve toward the median value after comparing the peak with the median value.

Using these criteria, total bone volume (BV) and trabecular bone volume (TBV) were obtained, and the cortical bone volume (CBV) was determined as the difference. The trabecular bone volume ratio (TBr; TBV/BV) was calculated using these numerical values. The average thickness of the skull through which ultrasound rays pass through (thickness) was calculated with the ExAblate system.

#### Procedure-Related Variables

The details of the procedure have been described previously. The intensity of the BBB opening (dose, AU) was calculated by monitoring the bubble activity during sonication with hydrophones embedded in the system and accumulating bubble activity of the subharmonic broadband from 75 to 155kHz, 80 kHz around half of the ultrasound frequency. The dose and energy delivered during sonication was obtained from the ExAblate 4000 system. Using these two values, the dose per 1 J of energy (D/E; dose/delivered energy, AU/J) was analyzed as an indicator of the ultrasonic energy transfer efficiency.

## Data Analysis

Statistical analysis was performed to identify the skull-related variables affecting ultrasonic energy transfer efficiency. All statistical analyses were performed using R version 4.0.1. Linear regression methods were used to determine the correlation between skull-related variables and ultrasonic energy transfer efficiency. All statistics were tested at significance level of 0.05.

## Results

### Results of Experimental Data

**FIGURE 3:**
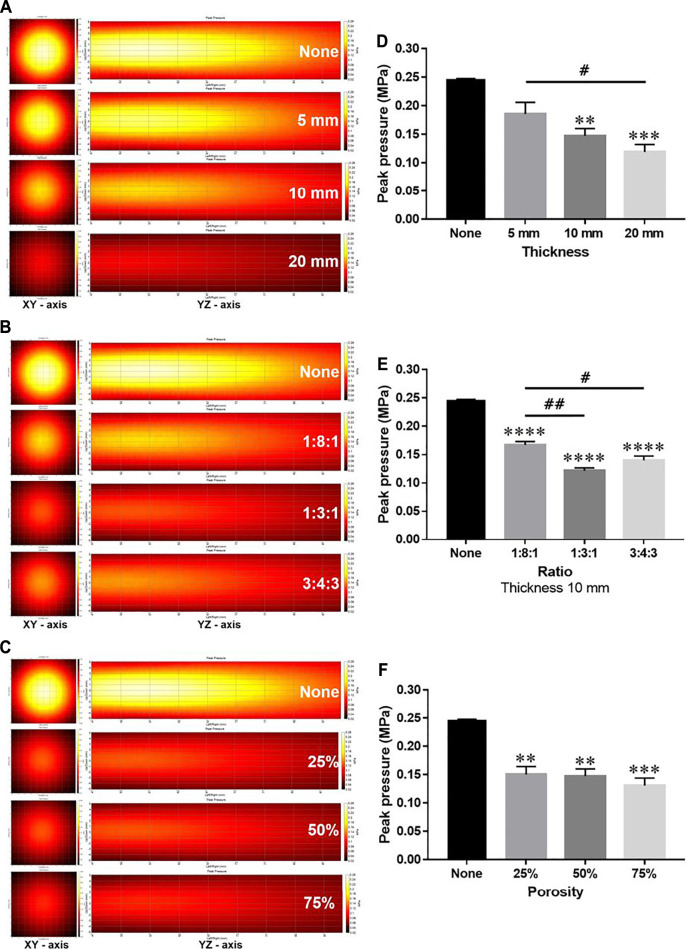
Comparison of ultrasound energy attenuation rates according to skull variables. **(A–C)** XY-axis and YZ-axis images of the ultrasound beam according to thickness, ratio, and porosity of the phantom. **(D–F)** Measurement results of peak pressure attenuation of ultrasonic energy according to thickness, ratio, and porosity. Data are shown as the mean ± standard error of the mean (SEM; *n* = 3 per group, one-way ANOVA with Tukey’s multiple comparison test). *p < 0.05 vs. None. #*p* < 0.05.

**FIGURE 4:**
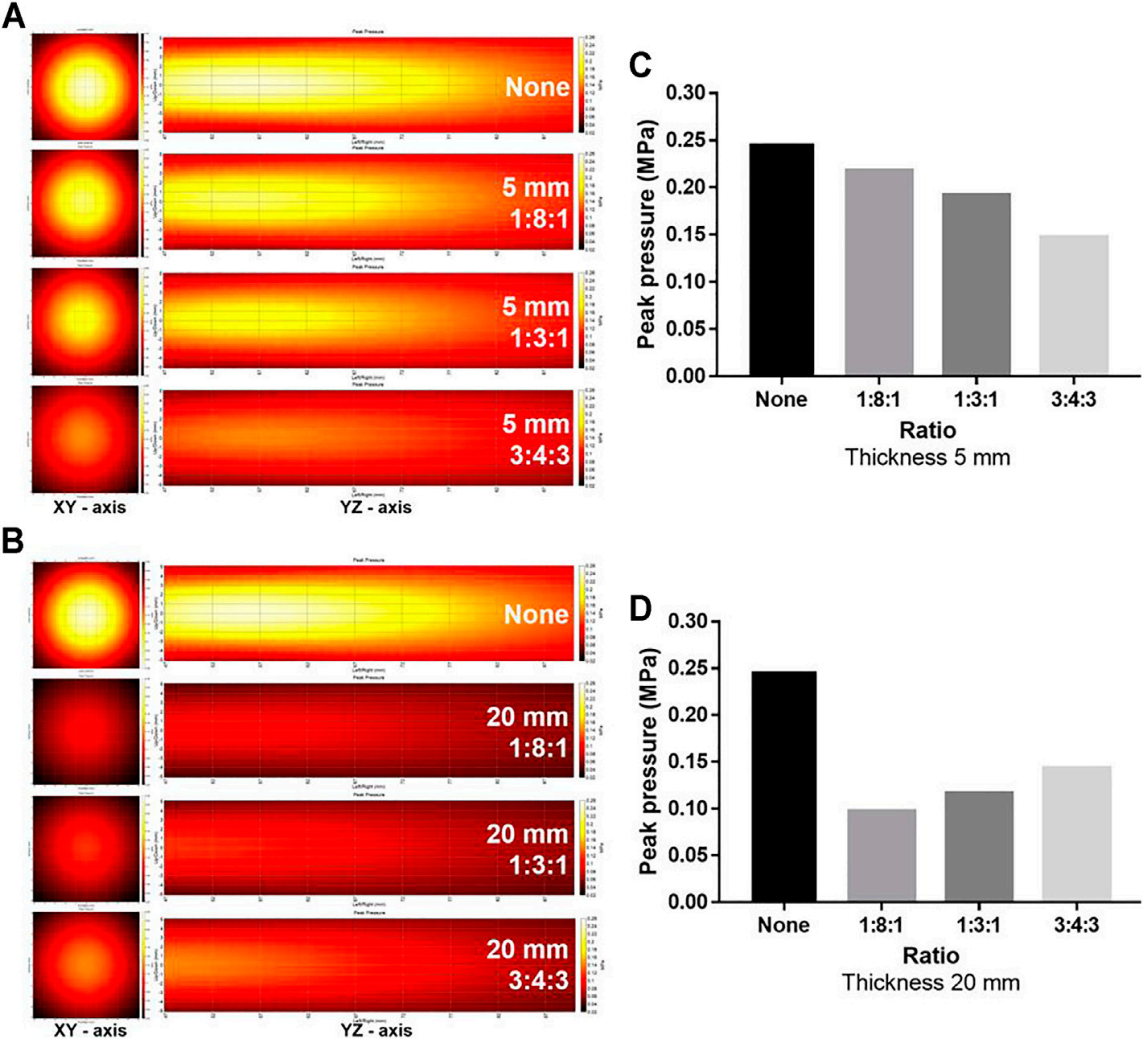
Comparison of ultrasound energy attenuation rates according to the ratio of the cortical bone to the trabecular bone. **(A, B)** XY-axis and YZ-axis images of the ultrasound beam according to thickness and ratio of the phantom. **(C, D)** Measurement results of peak pressure attenuation of ultrasonic energy according to the ratio and thickness. Comparison of ultrasound energy attenuation rates according to skull variables. (A–C) XY-axis and YZ-axis images of the ultrasound beam according to thickness, ratio, and porosity of the phantom (D–F).

Since the TBV and CBV were affected by the change in thickness and may influence the effect of thickness on the energy attenuation ratio, the attenuation ratio of the phantoms with a thickness of 5, 10, and 20 mm was averaged and compared (Table 2). Compared to controls, phantoms with 5 (0.185 MPa ± 0.020), 10 (0.147 MPa ± 0.012), and 20 mm (0.119 MPa ± 0.013) thickness showed significantly attenuated peak pressures (Figures 3A,D). Compared to controls, the 1:8:1 (0.167 MPa ± 0.005), 1:3:1 (0.121 MPa ±0.005), and 3:4:3 (0.140 MPa ± 0.007) ratio phantoms showed significantly attenuated peak pressures (Figures 3B,E). As the thickness of the phantom increased, the energy attenuation rate increased. However, there was no significant in peak pressures depending on the porosity (Figures 3C,F). Interestingly, as TBV is decreased, attenuation ratio has been gradually increased in the thickness of 5 mm (Figures 4A,C). On the other hand, although TBV is decreased, attenuation ratio has been decreased in the thickness of 20 mm (Figures 4B,D).

**TABLE 2:** Peak pressure of transmitted ultrasound energy in sixteen 3D printed skull phantom.

Phantoms	Peak pressure (MPa)	Attenuation ratio (%)
None	0.2469	0.00
0	0.1793	27.38
1	0.2187	11.42
2	0.1916	22.40
3	0.1476	40.22
4	0.1561	36.78
5	0.1116	54.80
6	0.1254	49.21
7	0.1705	30.94
8	0.1258	49.05
9	0.1477	40.18
10	0.1761	28.68
11	0.1274	48.40
12	0.1488	39.73
13	0.0973	60.59
14	0.1167	52.73
15	0.1434	41.92

### Results of Clinical Data

The average SDR was 0.39 ± 0.06, and the average thickness was 7.42 ± 1.28. The average TBV and BV were 147.44 ± 20.12 and 286.32 ± 29.67, respectively, and the average TBr was 0.52 ± 0.08. The average delivered energy was 34.0 ± 14.54, and the average BBB opening dose was 6.44 ± 3.32.

Among the skull-related variables, there was no correlation between TBV and BV (*p* = 0.55). However, there was a correlation between TBV and TBr (*p* < 0.001, *r* ^2^ = 0.5984) and TBr and BV (*p* < 0.001, *r* ^2^ = 0.3028), and these three variables were also correlated with CBV (BV, *p* < 0.001, *r* ^2^ = 0.6557; TBV, *p* = 0.002, *r* ^2^ = 0.2517; TBr, *p* < 0.001, *r* ^2^ = 0.8738). Thickness only correlated with BV (*p* = 0.011, *r* ^2^ = 0.1697), and SDR correlated with TBV but not with TBr (TBV, *p* = 0.021, *r* ^2^ = 0.1433; TBr, *p* = 0.094).

**FIGURE 5:**
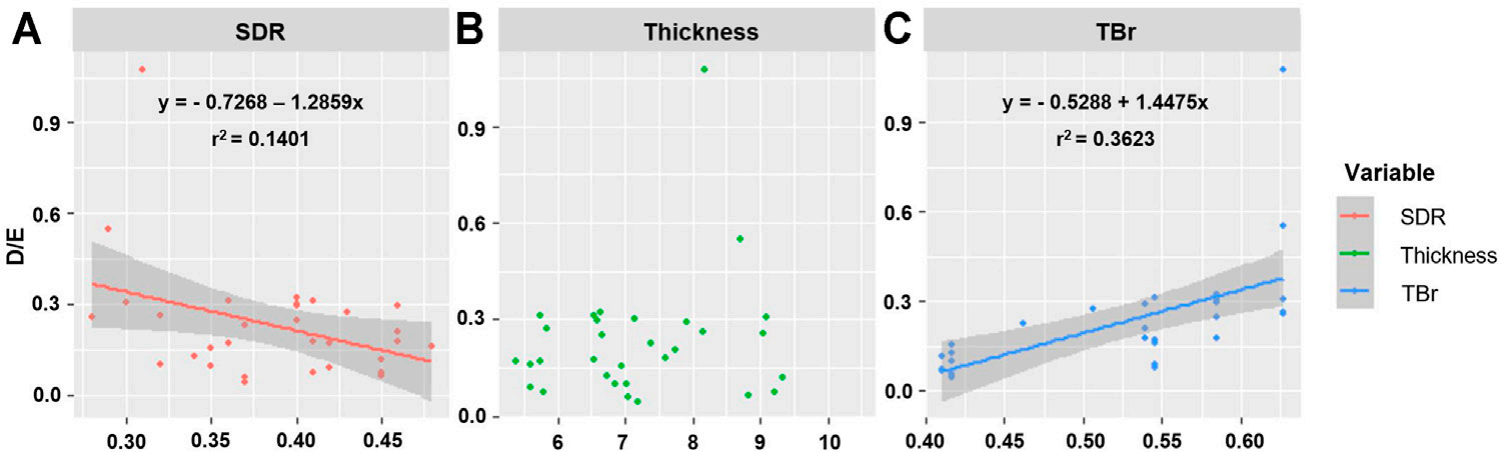
Representation of correlation between D/E and skull related factors. **(A)** SDR, **(B)** Thickness, **(C)** TBr.

Since BV, TBV, CBV, and TBr correlated with each other, and BV and TBV were related with SDR and thickness, respectively, the correlation with D/E was confirmed using SDR, thickness, and TBr to determine the influence of skull-related variable on BBB opening. SDR was negatively correlated with D/E, but had a small effect (*p* = 0.038, *r* ^2^ = 0.1401, y = −0.7268-1.2859x). TBr was positively correlated with D/E (*p* < 0.001, *r* ^2^ = 0.3623, y = -0.5288 + 1.4475x). Thickness did not correlate with D/E (*p* = 0.291). When patients were divided into two groups based on TBr value (0.5 vs. > 0.5), SDR and thickness had no significant correlation with D/E (SDR, *p* = 0.021, *r* ^2^ = 0.2501; thickness, *p* = 0.039, *r* ^2^ = 0.2012). However, in the group with a TBr <0.5, neither variables correlated with D/E (SDR, *p* = 0.4942; thickness, *p* = 0.607) (Figure 5; Table 3).

**TABLE 3:** Correlation of D/E with each factor in groups with SDR above 0.5 and below, thickness above 0.74 and below, and TBr above 0.5 and below.

	SDR <0.4	SDR ≥0.4	Thickness <0.74	Thickness ≥0.74	TBr <0.5	TBr ≥0.5
Number of patients	16	21	20	17	14	23
SDR	-	-	*p* = 0.276	*p* = 0.068	*p* = 0.917	*p* = 0.024
Thickness	*p* = 0.412	*p* = 0.361	-	-	*p* = 0.160	*p* = 0.041
TBr	*p* = 0.010	*p* = 0.004	*p* = 0.002	*p* = 0.045	-	-

## Discussion

FUS was first applied for CNS diseases in 2010, and it was thermal ablation using high-intensity energy in functional diseases (Martin et al., 2009; McDannold et al., 2010). After successful results, ultrasound treatment for CNS diseases has advanced one step further and FUS has more widely been applied to CNS diseases with low complication rates and high success rates. However, there were several cases where the temperature failed to rise sufficiently to create a lesion (McDannold et al., 2010; Chang et al., 2015). Based on these experiences, the concept of SDR, the ratio of the highest HU to the lowest HU, was introduced (Chang et al., 2015). In addition to SDR, studies on other skull variables that affect energy transmission have also been conducted, and these results are being used for patient selection (Chang et al., 2019; Jung et al., 2019). Using this criteria for patient selection, success rates have dramatically increased, and for each patient, it became possible to predict the thermal ablation temperature which has led to increased treatment efficiency.

While previous clinical studies with FUS involved ablation treatment using high-intensity energy, recent clinical studies have focused on modulating the BBB using low-intensity energy with microbubbles (Park et al., 2020). The BBB has posed a major challenge in the development of drugs targeting the CNS due to issues related to therapeutic agent delivery since it imposes size and biochemical restrictions on the passage of molecules (Pandey et al., 2016). However, it was discovered that BBB opening was possible with microbubble-mediated FUS, allowing for the delivery of drugs, which previously faced limitations in permeability, to the CNS (Ting et al., 2012). Therefore, many studies on BBB opening in CNS diseases are currently in progress.

FUS for thermal ablation uses high-intensity energy, while FUS for BBB opening uses low-intensity energy. The ultrasound wave used in these two types of FUS is different. Also, there is a difference in the attenuation rate when low- and high-intensity energies pass through a medium such as the skull. The data on variables influencing high-intensity FUS used for thermal ablation have been accumulated over several years (Chang et al., 2015; Jung et al., 2019), whereas the data on influencing factors for BBB opening are still limited. Therefore, for effective BBB opening, we tried to investigate the skull variables affecting the energy transmission of low-intensity FUS. In this study, we identified the variables affecting energy transmission in low-intensity FUS using 3D-printed phantoms, and we compared our findings with those of a BBB opening clinical study to confirm the relationship in practice.

SDR, which was thought to have a large effect on energy transmission in high-intensity FUS, had little effect on low-intensity FUS. Experimental data controlling other factors showed a slight influence on energy transmission. In the clinical data, although there is relevant, *r* ^2^ for D/E of SDR was 0.1401, which means the influence was small and, rather contrary to the experimental data, SDR and D/E were negatively related.

Thickness, which showed a significant effect on energy transmission in the experimental data, has little effect on D/E in the clinical data. In the experimental data, the thickness of the phantoms was set at 5, 10, and 20 mm, whereas patient skull thickness did not show a significant difference with a minimum value of 5.4 mm and maximum value of 10 mm. This may explain why the thickness does not show a correlation with D/E in the clinical dataset.

Instead, TBr showed considerable influence on energy transmission in the clinical data. TBr showed a significantly greater correlation with D/E than that of thickness and SDR. This suggests that TBr plays a very important role in low-intensity FUS. As the TBr increased, the D/E increased, and thus, the higher the ratio of the trabecular bone, the better the energy transfer for BBB opening. However, when subdivided, the effect of TBr was smaller in the low-thickness group compared with that in the high-thickness group. As the *β* value of TBr in the low-thickness group was lower than that in the high-thickness group, it can be confirmed that the experimental data in which the effect of CBV was greater than that of TBV when the thickness was thin is similarly applied.

Not all clinical data can be described as well as uniform results of experimental data. In patients with a TBr ≥0.5, SDR negatively correlated with energy transmission, while thickness positively correlated with energy transmission. This result is contrary to the experimental results. This part will need further confirmation based on more clinical data in the future.

There are several limitations. First, the quality of the phantoms produced through 3D printing has not been verified. Recently, 3D printing technology development has accelerated, so 3D-printed skulls are actually used in clinical practice. However, the artificial skull has limitations in realizing the detailed structure of the cortical and trabecular bones. Most of the artificial bones used in clinical practice are developed to replace the structure and function of bones. Currently, there is no material that can be similarly imitating the skull by reflecting all the detailed properties of bone (Kashte et al., 2017). We focused on the factors that affect energy attenuation in low-intensity FUS, and tried to investigate the effects of thickness, density, and TBV, CBV, and TBr factors on FUS energy. So, we tried to mimic the skull structurally as much as possible, but the material of the phantoms used in this study cannot be said to completely match the bone. Second, since the real trabecular bone is not uniform like the phantom, there is a limit to the applicability of laboratory data to the clinical field. Third, there is limited clinical FUS-mediated BBB opening data as most studies are currently in the clinical trial stage. The results of phantom analysis and clinical analysis are not perfectly matched, and further study with large number of data will be needed. However, as clinical FUS-mediated BBB opening research is starting all over the world, we think it will be of great help to clinical applications.

## Conclusion

Attenuation of ultrasound energy through the skull occurs during transcranial FUS-mediated BBB opening. In the current study, we tried to identify the factors affecting energy transmission during BBB opening with low-intensity FUS. As expected from the phantom study results, the attenuation of FUS energy increased with the thickness of the skull. Interestingly, there was no significant difference in the density of TB and the energy attenuation rate, but the TBV changed the energy attenuation rate according to the thickness of the skull, which can be considered as one of the important keys for the energy attenuation of TBr. Likewise, our clinical results indicate that TBr is an important factor related to the intensity of BBB opening. This information is helpful for patient and parameter selection for effective BBB opening.

## Data Availability

The raw data supporting the conclusions of this article will be made available by the authors, without undue reservation.

## Supplementary Material

Supplementary Figure S1 TIllustration of two cases. (A) D/E (the bubble activity per 1 J of energy) of a patient with an SDR of 0.29 and TBr of 0.63 was 0.55 AU/J. (B) D/E of a patient with an SDR of 0.37 and TBr of 0.42 was 0.04 AU/J.
