# Supplementary figures and images for: Factors Associated with Energy Efficiency of Focused Ultrasound Through the Skull: A Study of 3D-Printed Skull Phantoms and Its Comparison with Clinical Experiences
Source: Front Bioeng Biotechnol. 2021 Dec 10;9:783048. doi: 10.3389/fbioe.2021.783048 (PMC8708563; doi:10.3389/fbioe.2021.783048)

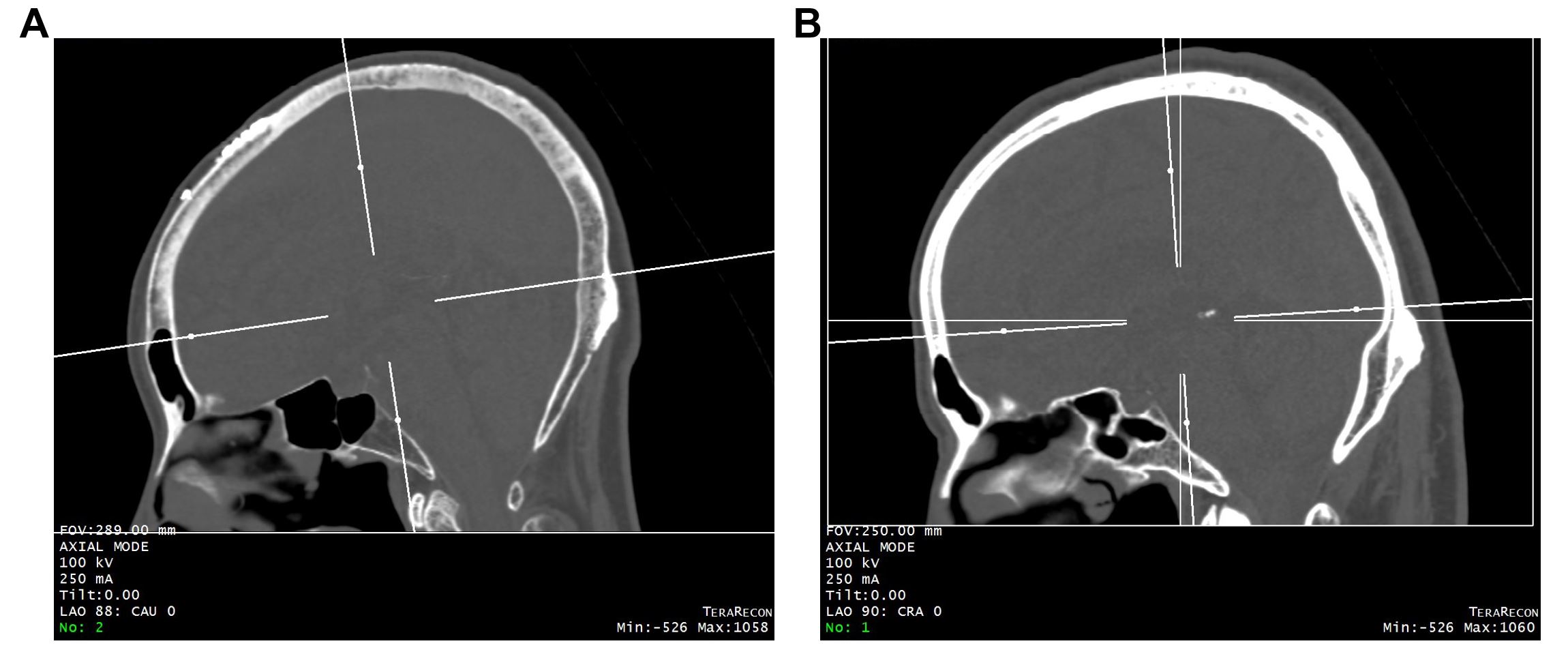

Supplement: Supplementary file 1 [file Image1.JPEG]
